# Clinical Outcomes Following Diathermy Versus Scalpel Intraoral Incisions in Mandibular Fracture Surgery: A Randomized Comparative Analysis

**DOI:** 10.7759/cureus.114061

**Published:** 2026-08-06

**Authors:** Ambuj Aggarwal, Atul Sharma, Dinesh Kumar, Gurinder Thind, Devyani Bahl

**Affiliations:** 1 Craniomaxillofacial Surgery, Amrita Institute of Medical Sciences and Research Centre, Kochi, IND; 2 Oral and Maxillofacial Surgery, Maharishi Markandeswar College of Dental Sciences, Maharishi Markandeshwar (Deemed to Be University), Mullana, IND; 3 Oral Surgery, Maharishi Markandeswar College of Dental Sciences, Maharishi Markandeshwar (Deemed to Be University), Mullana, IND; 4 Oral and Maxillofacial Surgery, Baba Farid University of Health Sciences, Sunam, IND; 5 Oral and Maxillofacial Surgery, Private Consultant, Mumbai, IND

**Keywords:** blood loss, diathermy, incision, mandibular fracture, scalpel

## Abstract

Mandibular fracture surgery requires intraoral incisions, and the choice between scalpel and diathermy may affect operative efficiency, blood loss, pain, and healing.

Aim

To compare and evaluate the outcome of intraoral incisions with the use of diathermy and a scalpel in the treatment of mandibular fractures.

Settings and design

Prospective randomized comparative study conducted at a single tertiary care center.

Methodology

Thirty patients with mandibular fractures were enrolled and randomized into two groups (15 per group). Group A underwent incision with a scalpel, and Group B underwent incision with diathermy. Incision time, blood loss, total operating time, postoperative pain, and wound healing were evaluated at 24 hours and on postoperative days three, seven, and 10.

Statistical analysis

Data were analyzed using SPSS version 21.0 (IBM Corp., Armonk, USA). Normality of continuous variables (age, incision time, blood loss, total operating time, postoperative pain scores, and wound healing scores) was assessed using the Shapiro-Wilk test; normally distributed variables were compared using an unpaired t-test and non-normally distributed variables using the Mann-Whitney U test. Categorical variables were compared using the chi-square or Fisher's exact test, as appropriate. A p value < 0.05 was considered statistically significant. Sample size was based on convenience sampling without a priori power calculation.

Results

Thirty patients with mandibular fractures were enrolled and randomized into two groups. The mean age was 25.87 ± 10.63 years in the scalpel group and 26.80 ± 8.00 years in the diathermy group, with no significant difference between groups. Male patients comprised 14/15 (93.3%) in each group. The mean incision time was 239.5 ± 37.95 seconds in the diathermy group and 248.0 ± 32.72 seconds in the scalpel group. Mean blood loss was 6.2 ± 5.88 mL and 8.5 ± 6.4 mL, respectively. Total operating time was 94.47 ± 37.35 minutes in the diathermy group and 80.47 ± 30.33 minutes in the scalpel group. Postoperative pain was significantly higher with diathermy at the early follow-up visits, whereas wound healing outcomes were comparable in both groups at all time points.

Conclusions

In mandibular fracture surgery, diathermy may modestly reduce incision-related bleeding and time, though this study was underpowered to confirm these trends; scalpel incisions were associated with significantly less early postoperative pain, with comparable wound healing outcomes between techniques. Larger, adequately powered trials are needed to confirm the hemostatic and efficiency trends observed.

## Introduction

Maxillofacial trauma cases have seen a massive upsurge in recent years owing to the increased number of road accidents due to increased vehicle use, overspeeding, and traffic violations. Additional contributing factors may include interpersonal violence, pathological conditions, firearm accidents, occupational injuries, and/or sports-related trauma [1]. Mandibular fractures are the second most common type of fracture seen in the orofacial region. Amongst these, the incidence of fractured sites varies as parasymphysis (40.3%), angle (28.8%), condyle (27.6%), symphysis (12.5%), and coronoid process (4.3%). However, when alterations are involved, angle fractures are the most fractured site [2].

The choice of incision for an oral and maxillofacial procedure typically remains at the surgeon’s discretion. For mandibular fractures requiring open reduction and internal fixation, the intraoral approach is generally preferred over the extraoral route, as it avoids visible facial scarring and risk of facial nerve injury while carrying a comparable risk of postoperative infection [3]. Traditionally, mucosal incisions were made by stainless steel scalpels, as they were easy to use, more accurate, and caused less damage to the oral soft tissues. However, they are often associated with increased bleeding and postoperative pain [4]. To overcome these challenges, surgical diathermy emerged as an effective alternative. While electrosurgical instruments have been studied extensively for skin incisions, fewer studies have evaluated their use for intraoral mucosal incisions. Hence, the present study was conducted to compare the mucosal incisions given with a scalpel and diathermy for mandibular fractures at any anatomical site requiring an intraoral vestibular approach. The primary objective of this study was to compare intraoral vestibular incision time and intraoperative blood loss between diathermy and scalpel techniques in patients undergoing open reduction and internal fixation for mandibular fractures. Secondary objectives included comparison of total operating time; postoperative pain (via visual analog scale) at 24 hours and postoperative days three, seven, and 10; wound healing (via Southampton grading) at the same time points; operator comfort; and postoperative complications.

For consistency, we use 'diathermy' throughout to refer to the electrosurgical technique used in this study; where cited literature specifically uses 'electrocautery,' this terminology is retained to accurately reflect the source.

## Materials and methods

Study design

This prospective study was conducted on a total of 30 patients with mandibular fractures reporting to the outpatient department of oral and maxillofacial surgery over a span of three years, who were indicated for open reduction and internal fixation (ORIF). Institutional Ethics Committee approval was obtained. The study adhered to the Declaration of Helsinki. All patients provided written informed consent.

Participants

Thirty consecutive patients with mandibular fractures requiring open reduction and internal fixation (ORIF) via intraoral vestibular incision were enrolled. Simple randomization was performed by an independent research assistant using a coin toss, conducted individually immediately before each procedure (heads: scalpel Group A; tails: diathermy Group B), allocating 15 fracture sites per group. The toss was performed without a witness present, and no formal allocation concealment mechanism (e.g., sealed opaque envelopes) or block randomization was used; the operating surgeon was informed of the allocation only at the point of incision. A single experienced surgeon (blinded to allocation until incision) performed all procedures to minimize inter-operator variability.

Eligibility criteria are summarized in Table 1. Patients older than 18 years with American Society of Anesthesiologists (ASA) I or II status who required open reduction and internal fixation through an intraoral vestibular incision were included, whereas patients with ASA III-VI status, deleterious habits such as smoking or alcohol consumption, non-compliance with the study protocol, or intraoral laceration, wound, or ecchymosis at the planned incision site were excluded.

**Table 1:** Eligibility criteria for study participants ASA: American Society of Anesthesiologists.

No	Criterion
Inclusion	
1	Age > 18 years
2	ASA I or II status
3	Mandibular fracture requiring open reduction and internal fixation through an intraoral vestibular incision
Exclusion	
1	ASA III, IV, V, or VI status
2	Smoking or alcohol consumption
3	Non-compliance with study protocol
4	Intraoral laceration or wound at the planned incision site
5	Ecchymosis at the planned incision site

Surgical procedures/interventions

All patients underwent a routine pre-anesthetic checkup, including standard blood workup and airway assessment, to confirm fitness for general anesthesia and to reconfirm ASA status before surgery.

**Figure 1:**
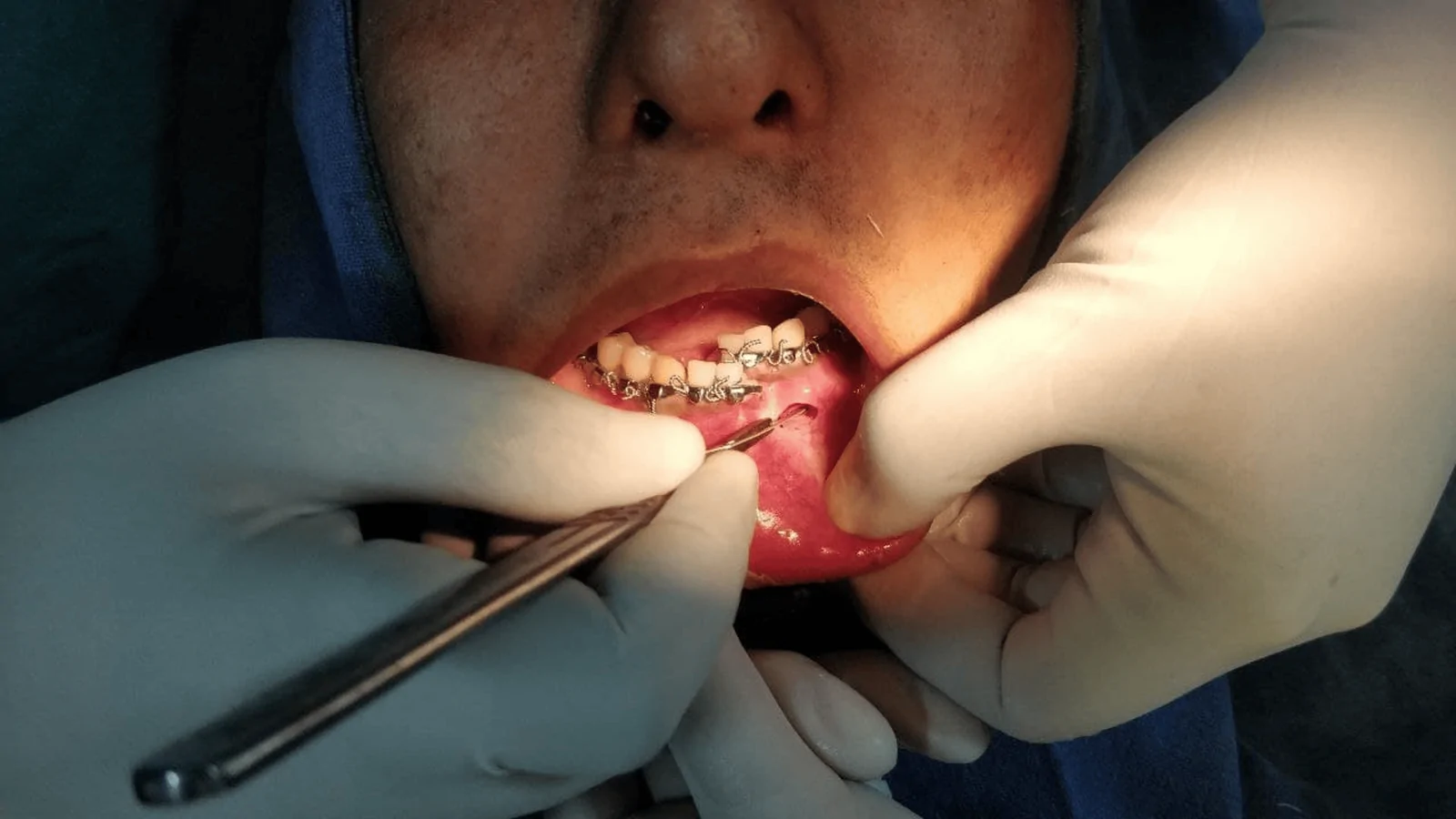
Intraoral incision with a scalpel in Group A

**Figure 2:**
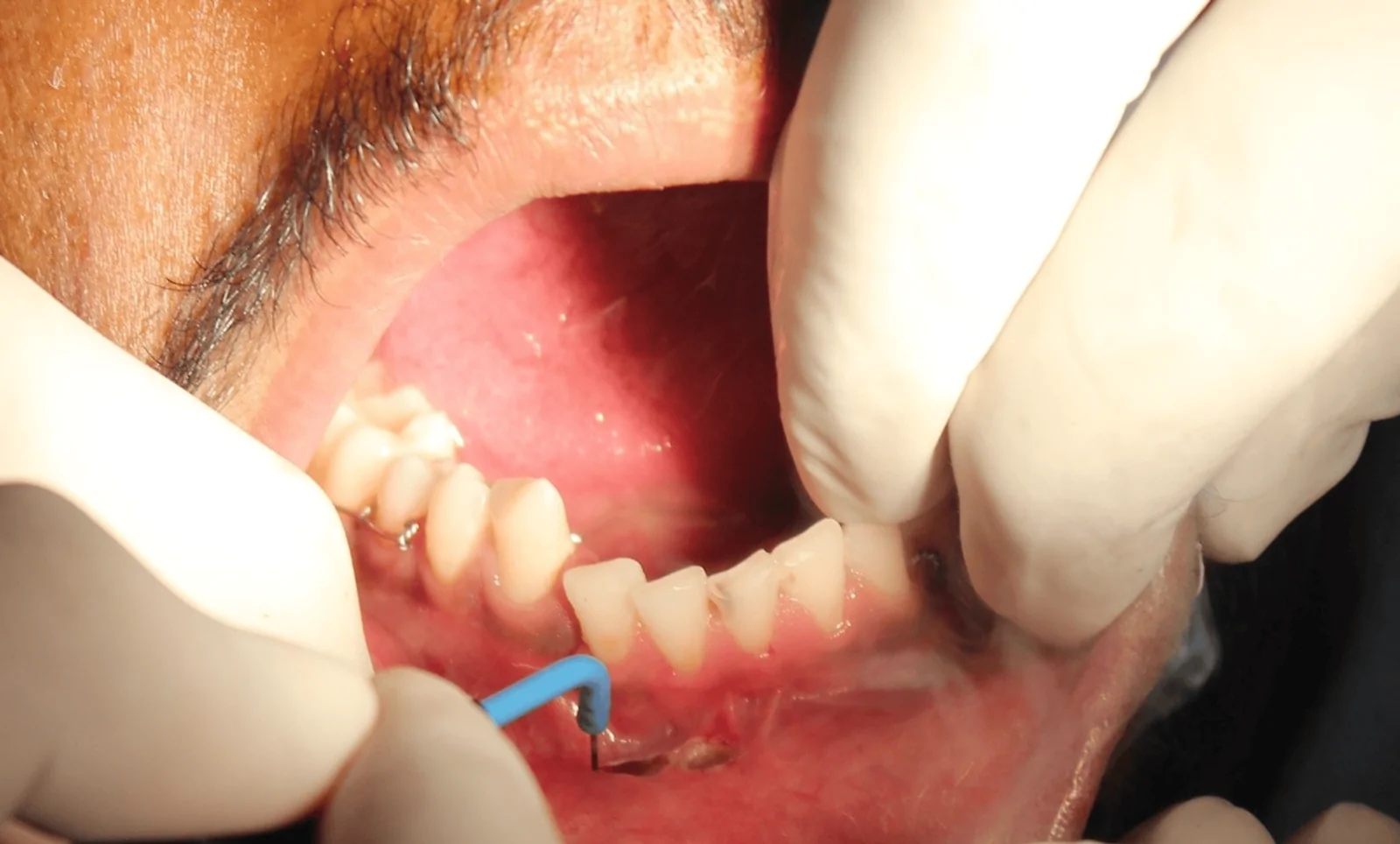
Intraoral incision with a diathermy in Group B

**Figure 3:**
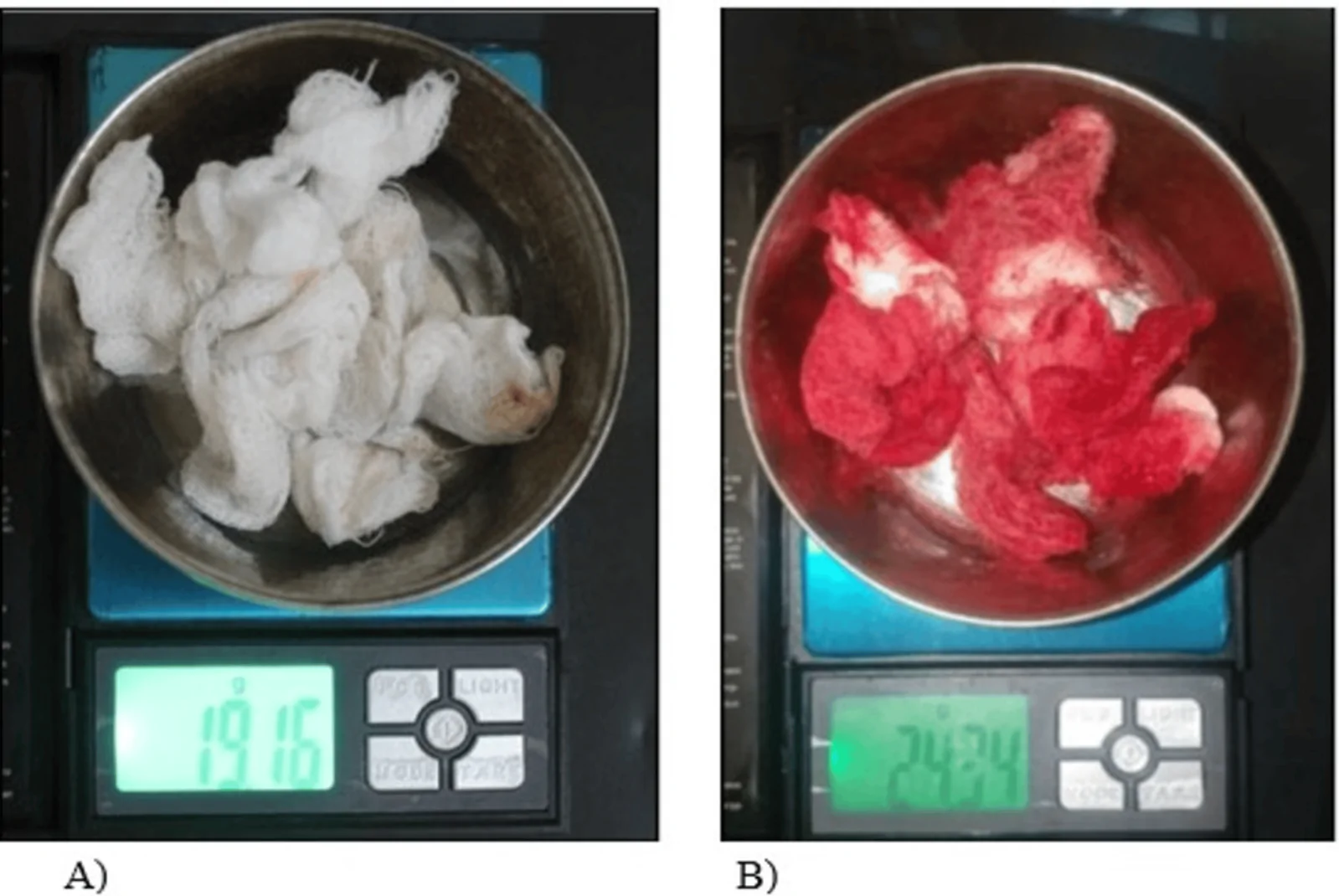
A) Pre-operative weight (saline soaked gauze pieces) B) Post-operative weight (blood soaked gauze pieces)

All the cases in this study were operated on by a single surgeon under general anesthesia. Standard surgical procedures were followed for both groups. Before starting the procedure, painting and draping were done under all aseptic conditions using standard aseptic protocol. Local anesthesia with adrenaline (1:200000) was administered as a classic inferior alveolar nerve block. Approx. 2 ml of lignocaine 2% with adrenaline (1:200000) was also given as local infiltration at the surgical site. In Group A, an incision was made using a stainless steel scalpel [Bard-Parker handle no. 3 (Orthomax, Vadodara, India) and blade no. 15 (Niraj Industries, Faridabad, India)] (Figure 1), and in Group B, an incision was made using diathermy (Art electron®, New Taipei City, Taiwan) (Figure 2). An intraoral vestibular incision was given, and a full-thickness mucoperiosteal flap was raised. The duration of the incision was noted from the start of the incision till the exposure of the fracture site. Blood loss was quantified gravimetrically: gauze pieces were pre-soaked in 0.9% normal saline and pre-weighed using an electric weight measurer. During the procedure, only gauze pieces (no suction) were used to soak blood until exposure of the fracture site; in the diathermy group, suction was used solely to evacuate electrosurgical fumes, not blood. Gauze pieces were reweighed post-exposure, and blood loss was calculated using a standard 1 g = 1 mL conversion (Figure 3). The manipulation of the fracture segments was done, anatomic reduction was achieved, and fixation was done using titanium miniplates. Hemostasis was achieved, and the wound was closed. Postoperative care included amoxicillin-clavulanate 625 mg three times daily for five days, ibuprofen 400 mg as needed for pain, chlorhexidine mouth rinses, a soft diet, and follow-up visits at least weekly for a minimum of 10 days.

Outcome measures

The primary outcomes were incision time, defined as the interval from blade or diathermy contact to exposure of the fracture site, and intraoperative blood loss (measured gravimetrically as described in the surgical procedures/interventions section).

The secondary outcomes were total operating time; postoperative pain assessed using a 100 mm visual analog scale on postoperative days one, three, seven, and 10; wound healing assessed by two independent blinded observers using the Southampton grading system; and postoperative complications, including dehiscence, infection, and plate exposure if present. Wound dehiscence and plate exposure were diagnosed by visible clinical examination (wound margin separation for dehiscence; visible hardware exposure through the mucosa for plate exposure), without adjunctive imaging. Infection was assessed clinically, based on the absence of purulent discharge, erythema, or fever; no wound cultures were obtained. Operator comfort was rated qualitatively by the operating surgeon immediately after each procedure as 'Good' or 'Moderate,' based on subjective impression of ease of tissue handling, visibility, and bleeding interference during the incision. No validated or standardized operator comfort scale was used.

Statistical analysis

Data were analyzed using SPSS version 21.0 (IBM Corp., Armonk, USA). Normality was tested using the Shapiro-Wilk test. Continuous variables were presented as mean ± SD, and an unpaired t-test (normal) or Mann-Whitney U (nonparametric) was used. Categorical variables were tested with chi-square/Fisher's exact test. p < 0.05 was considered statistically significant. Sample size was determined by convenience sampling (n = 30; 15 per group) rather than a priori power calculation. Post-hoc power analysis based on observed effect sizes indicated the study was underpowered for the primary outcomes (power ≈ 10% for incision time; power ≈ 16% for blood loss).

## Results

Demographics

Thirty patients underwent treatment for mandibular fractures requiring intraoral vestibular incision for open reduction and internal fixation, with 15 patients allocated to each group. The mean age in the scalpel group was 25.87 ± 10.63 years and in the diathermy group was 26.80 ± 8.00 years, with no statistically significant difference between groups (Table 2). Males constituted 14/15 (93.3%) patients in both the scalpel and the diathermy groups (Table 3).

**Table 2:** Age distribution of the study groups

Group	N	Mean ± SD	Mean Difference (95% CI)*	t value	p value
Scalpel	15	25.87 ± 10.63	-0.93 (-7.93, 6.07)	-0.272	0.788
Diathermy	15	26.80 ± 8.00			

**Table 3:** Gender distribution between the study groups Values are presented as n (%). Categorical comparisons were analyzed using the chi-square test; effect size is reported as Cramér’s V

Variable	Group A (Scalpel) n (%)	Group B (Diathermy) n (%)	Test statistic	df	p value	Effect size
Male	14 (93.3)	14 (93.3)	0.000	1	1.000	0.000
Female	1 (6.7)	1 (6.7)	0.000	1	1.000	0.000

**Figure 4:**
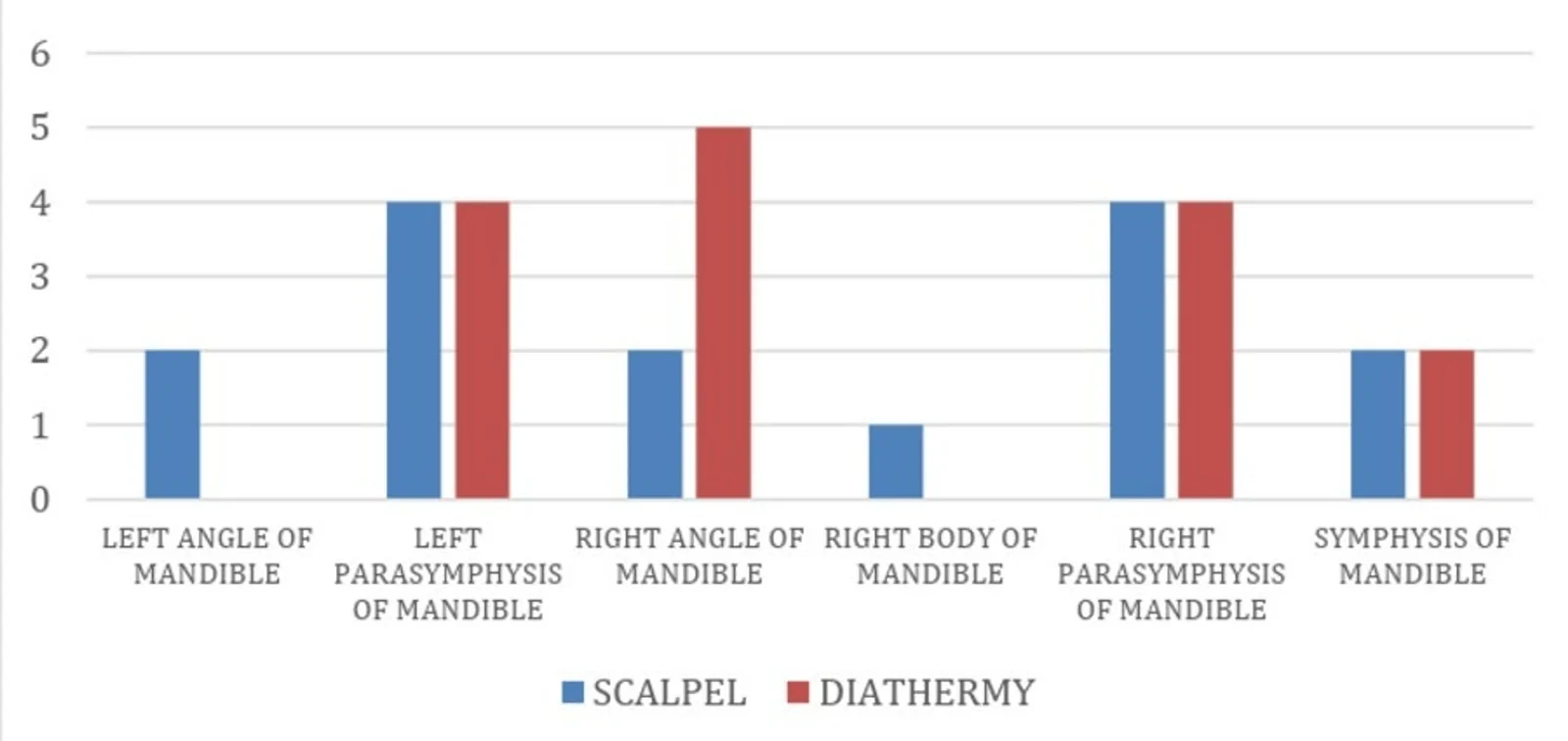
Distribution of surgery site between groups

The left and right parasymphysis regions of the mandible were the commonest sites of surgery in this study. There was no statistically significant difference in the surgery site between groups p = 0.218 (Figure 4).

Study parameters

The mean incision time was 239.5 ± 37.95 seconds in the diathermy group and 248.0 ± 32.72 seconds in the scalpel group, while mean blood loss was 6.2 ± 5.88 mL and 8.5 ± 6.4 mL, respectively. Although both measures favored diathermy, the differences were not statistically significant. Total operating time was 94.47 ± 37.35 minutes in the diathermy group and 80.47 ± 30.33 minutes in the scalpel group, again without a significant difference (p = 0.269) (Table 4).

**Table 4:** Comparison of operative parameters between scalpel and diathermy groups Values expressed as mean ± standard deviation (SD). An independent samples t-test was used for comparison. A p value of < 0.05 was considered statistically significant. All mean differences and t-values are calculated as Scalpel minus Diathermy.

Parameter	Scalpel (N = 15) Mean ± SD	Diathermy (N = 15) Mean ± SD	Mean Difference (95% CI)*	t value	p value
Duration of incision (sec)	248.00 ± 32.72	239.53 ± 37.95	-8.47 (-35.00, 18.05)	-0.654	0.518
Blood loss (mL)	8.51 ± 6.40	6.22 ± 5.88	-2.29 (-6.89, 2.31)	-1.020	0.316
Total operating time (min)	80.47 ± 30.33	94.47 ± 37.35	14.00 (-11.44, 39.44)	1.127	0.269

In the scalpel group, 2 (13.3%) of cases were associated with moderate operator comfort, whereas in the diathermy group, 4 (26.7%) of cases showed moderate operator comfort. There was no statistically significant difference in operator comfort between the groups (p = 0.361) (Table 5).

**Table 5:** Assessment of operator comfort between groups Fisher's exact test used given an expected cell count below 5.

	Good n (%)	Moderate n (%)	Total	Test	df	p value
Scalpel	13 (86.7)	2 (13.3)	15	Fisher's exact	1	0.361
Diathermy	11 (73.3)	4 (26.7)	15			

Postoperative pain scores were significantly lower in the scalpel group compared to the diathermy group at 24 hours, third day, and seventh day (p < 0.05). However, by the 10th day, the difference was not statistically significant (p = 0.623) (Table 6).

**Table 6:** Comparison of pain scores between scalpel and diathermy groups at different time intervals Values expressed as mean ± standard deviation (SD). An independent samples t-test was used for comparison. A P < 0.05 was considered statistically significant. Mean difference and t-value calculated as Scalpel minus Diathermy.

Time Point	Scalpel (Mean ± SD)	Diathermy (Mean ± SD)	Mean Difference (95% CI)*	t-value	p value
After 24 hours	4.80 ± 0.94	7.00 ± 1.07	-2.20 (-2.95, -1.45)	-5.982	< 0.0001
On 3rd day	3.07 ± 1.03	4.67 ± 0.98	-1.60 (-2.35, -0.85)	-4.361	< 0.0001
On 7th day	1.40 ± 1.06	2.53 ± 0.74	-1.13 (-1.81, -0.45)	-3.400	0.002
On 10th day	0.47 ± 0.74	0.33 ± 0.72	0.14 (-0.44, 0.72)	0.498	0.623

Healing scores were comparable between the scalpel and diathermy groups at all time points, with no statistically significant differences observed (p > 0.05) (Table 7).

**Table 7:** Comparison of Southampton healing scores between scalpel and diathermy groups Values expressed as mean ± standard deviation (SD). An independent samples t-test was used for comparison. A p < 0.05 was considered statistically significant. Mean difference and t-value calculated as Scalpel minus Diathermy.

Time Point	Scalpel (Mean ± SD)	Diathermy (Mean ± SD)	Mean Difference (95% CI)*	t-value	p value
After 24 hours	2.07 ± 0.88	2.07 ± 0.59	0.00 (-0.56, 0.56)	0.000	1.000
On 3rd day	1.73 ± 0.88	1.87 ± 0.74	-0.14 (-0.78, 0.50)	-0.447	0.658
On 7th day	1.00 ± 0.65	1.27 ± 0.88	-0.27 (-0.86, 0.32)	-0.939	0.356
On 10th day	0.60 ± 0.63	0.73 ± 0.96	-0.13 (-0.72, 0.46)	-0.449	0.657

A total of four cases of wound dehiscence were observed, comparable between the groups (13% each), all of which healed by secondary intention; one case in the diathermy group showed transient plate exposure. No infections or readmissions were reported (Table 8).

**Table 8:** Postoperative complications Values are presented as n (%). Categorical comparisons were analyzed using the chi-square test or Fisher’s exact test, as appropriate.

Complication	Group A (Scalpel) n (%)	Group B (Diathermy) n (%)	Test used	df	p value	Effect size
Dehiscence	2 (13.3)	2 (13.3)	Chi-square	1	1.000	0.000
Plate exposure	0 (0.0)	1 (6.7)	Fisher's exact	—	1.000	—

## Discussion

This prospective single-center randomized controlled trial systematically compared diathermy (electrosurgical unit) versus traditional stainless steel scalpel for intraoral vestibular incisions during open reduction and internal fixation (ORIF) of mandibular fractures. Primary endpoints focused on incision efficiency, including time to fracture exposure and intraoperative blood loss. Secondary outcomes encompassed total operating time; postoperative pain assessed by VAS; wound healing evaluated by blinded Southampton grading; and adverse events.

The study revealed nuanced trade-offs between the two techniques. Diathermy conferred modest intraoperative advantages in speed and hemostasis, though these trends did not reach statistical significance. However, diathermy also imposed significantly elevated early postoperative pain compared to a scalpel. By 10 days post-intervention, both techniques yielded clinically equivalent healing outcomes and demonstrated similarly low complication profiles.

Intraoperative efficiency: time and hemostasis

Incision duration trended shorter with diathermy (mean 239.5 ± 37.95 seconds) compared to scalpel (248 ± 32.72 seconds; p = 0.58, unpaired t-test). Blood loss similarly favored diathermy (6.2 ± 5.88 ml vs. 8.5 ± 6.4 ml; p = 0.39, Mann-Whitney U test). These findings align with the biophysical principles of electrosurgery [5]. High-frequency alternating current (0.3-5 MHz) induces intracellular frictional heating (60-100°C), sparking instantaneous cellular vaporization, steam explosions, and protein coagulation. This mechanism simultaneously transects tissue and seals microvasculature (< 0.5 mm diameter) through endothelial necrosis and vasoconstriction, rendering the surgical field drier without requiring suction. Our gravimetric blood loss protocol (described in Methods) captured this hemostatic advantage. These results resonate with domain-specific literature. Nagargoje et al. [6] conducted a strikingly parallel randomized trial published in Annals of Maxillofacial Surgery, demonstrating statistically robust diathermy superiority for anterior mandibular fractures. Their study showed significantly reduced time for incision and mucoperiosteal flap elevation (2.99 ± 0.60 min vs. 4.79 ± 1.0 min with a scalpel, p < 0.001) and decreased blood loss (7.91 ± 0.0 ml vs. 13.32 ± 2.5 ml, p < 0.001). Their restriction to anterior fracture sites likely amplified hemostatic gains in a confined, highly vascular zone. In contrast, our heterogeneous cohort (parasymphysis 53%, angle 27%, body 20%) introduced greater anatomical variability. This diversity, compounded by convenience sampling (n = 30 total, yielding post-hoc power of approximately 65-75% for primary outcomes), may explain why our results showed favorable trends without achieving statistical significance. Broader electrosurgical evidence supports these findings. Ly et al.'s systematic review and meta-analysis in general surgery demonstrated that diathermy halves skin incision blood loss and reduces operative time by 10-20% [7]. A more directly comparable population was examined by Sherpa et al., whose 2025 study of vestibular incisions for mandibular angle and body fractures similarly found a significant difference in incision time favoring electrocautery, lending further support to a fracture-site-dependent magnitude of hemostatic benefit [8]. Similar advantages have been documented in oral surgery, such as the study by Sharma and Sachdeva showing reduced flap elevation time with electrosurgery (5.13 ± 7.3 min) versus scalpel (6.55 ± 7.8 min) [9]. A more recent comparative study by Joty et al. similarly found shorter incision time and reduced blood loss with diathermy in elective general surgery, supporting the consistency of this hemostatic advantage across surgical settings [10]. This is further corroborated by the largest meta-analysis in this domain, Ismail et al.'s pooled analysis of 41 studies (6,422 participants), which confirmed significantly reduced blood loss, incision time, and operative time with electrocautery compared to scalpel, with no significant difference in wound infection rates [11].

Total operating time: a countervailing drawback

Despite faster incision times, diathermy paradoxically extended overall operating duration (94.47 ± 37.35 min vs. 80.47 ± 30.33 min for a scalpel; p = 0.22). This unexpected finding reflects a critical limitation of electrosurgical incisions. Diathermy produces a coagulum-eschar layer with lateral thermal contraction, creating a 1-3 mm zone of desiccation and necrosis. The resulting contracted, friable wound margins are prone to tearing during subsequent surgical steps, including flap reflection, fracture manipulation, and layered closure with 3-0 Vicryl suture. In contrast, scalpel incisions produce clean, non-traumatized edges that are more amenable to precise tissue handling. This difference in tissue quality impacts surgical efficiency during the most technically demanding portions of the procedure. Mucosal-specific investigations corroborate these observations. Liboon et al. (1997) [4] documented in porcine models that electrosurgery and CO₂ laser flaps were "difficult to manipulate" despite faster initial creation, with histologic evidence of charring that delayed tissue apposition. Similar findings with constant-voltage electrocautery demonstrated inferior tissue handling characteristics compared to blade incisions. In short, the same thermal mechanism that shortens incision time appears to lengthen subsequent operative steps by compromising tissue handling quality.

Postoperative pain: the toll of thermal injury

Diathermy markedly exacerbated early postoperative pain compared to a scalpel. VAS scores on day 1 post-surgery were significantly higher in the diathermy group (6.8 ± 1.4 vs. 4.2 ± 1.1, p < 0.0001), as were day three scores (5.4 ± 1.2 vs. 3.1 ± 0.9, p < 0.0001). However, pain levels converged by day seven (1.7 ± 0.8 vs. 1.5 ± 0.7, p = 0.45) and remained equivalent at day 10 (0.9 ± 0.5 vs. 0.8 ± 0.4, p = 0.67).

This temporal pain profile implicates collateral thermal spread as the primary mechanism. Monopolar diathermy generates 0.5-2.5 mm of lateral tissue necrosis, with the extent depending on power settings (typically 20-40W in cutting mode) [12]. This thermal injury denervates free nerve endings, induces localized ischemic hypoxia, and amplifies the release of nociceptive mediators, including prostaglandins, substance P, and interleukins IL-1 and IL-6. Animal models support this mechanism. Hasar et al. demonstrated in rat studies that electrosurgery induced prolonged pain lasting 1-7 days through elevation of acute-phase reactants and ischemic tissue injury analogous to the peritoneal damage described by Ten Broek et al. [13, 14].

The clinical literature presents heterogeneous findings that merit discussion. Interestingly, Nagargoje et al. [6] reported counterintuitively reduced pain in their diathermy group at 24 and 48 hours post-surgery. They attributed this to superior surgical field visualization minimizing iatrogenic trauma, though differences in postoperative analgesia protocols may also have contributed. Similarly, Kalia et al. [15] found no significant pain differences between techniques in impacted third molar surgery, possibly due to the thermal buffering capacity of keratinized gingiva compared to the thinner vestibular mucosa in our mandibular fracture cohort. Our study's uniform postoperative regimen (ibuprofen as needed, chlorhexidine rinses) helps isolate the technique-specific effects on pain.

Wound healing dynamics and complications

Southampton wound healing scores demonstrated clinical equivalence between techniques across all assessment time points. Day one scores averaged 2.1 ± 0.5 for diathermy versus 1.9 ± 0.4 for scalpel (p = 0.31), with similar patterns on day 3 (1.9 ± 0.4 vs. 1.7 ± 0.3, p = 0.22) and days seven and 10 (all p > 0.4). Scores represented the highest grade assigned by two blinded assessors. The predominance of Grade 1 (normal healing) and Grade 2 (mild bruising or erythema) reflects the favorable intraoral healing environment, characterized by abundant salivary flow and rich vascular supply.

The mechanisms underlying these equivalent healing outcomes differ by technique. Scalpel incisions preserve endothelial integrity and maintain normal perfusion gradients, as demonstrated by Manivannan et al. [16], who documented 30% superior gingival Doppler blood flow measurements at day seven and beyond with blade incisions. This preserved microcirculation fosters rapid fibroblast proliferation and organized collagen deposition. Conversely, diathermy achieves hemostasis through eschar formation, which effectively curtails serosanguineous discharge (evidenced by the rarity of Grade 3 findings in our study). However, this comes at the cost of delayed re-epithelialization due to collagen denaturation, consistent with early animal evidence from Pope et al., who first characterized impaired wound healing following electrosurgical incisions in a canine model [17]. Liboon's [4] histologic studies demonstrated exuberant granulation tissue formation persisting through weeks three to six following electrosurgical incisions, representing a prolonged inflammatory phase before epithelial maturation. This said, more recent best-practice evidence synthesis by Mansour et al. concluded that electrocautery incisions are cosmetically noninferior to scalpel despite this histologic difference, suggesting that the clinical impact of delayed re-epithelialization on long-term scar quality may be less significant than initial mechanistic concerns would predict [18].

Despite these mechanistic differences, clinical outcomes remained equivalent. Adverse events were balanced at 13% per treatment arm. The scalpel group experienced two wound dehiscences, while the diathermy group also had two complications, including one case of transient plate exposure. All adverse events were classified as minor, resolving via secondary intention healing without purulent discharge or signs of infection (no Grade 4 or 5 Southampton scores). Complete resolution occurred within 3 weeks in all cases, with no patients requiring reoperation or readmission.

These complication rates align with established literature. Wahab et al. [19] reported in a split-mouth trial design that scalpel incisions yielded fewer complications (p < 0.05), though their study examined different anatomical sites. Our findings fall within expected ranges for mandibular ORIF procedures generally, where dehiscence rates of 10-20% and infection rates below 5% are considered typical.

This study incorporates several methodological strengths that enhance the validity of its findings. The prospective randomized design, using coin toss allocation, ensures temporal sequence and minimizes selection bias, though stratification was not employed. Primary outcome measures were objective and reproducible: incision time was measured with a stopwatch, while blood loss was quantified gravimetrically using pre- and post-weighed gauze (1 g = 1 ml equivalence). Wound healing assessment employed independent blinded adjudication, with the highest score from dual assessors used to ensure conservative grading and minimize detection bias.

Limitations

Several methodological limitations should be considered. First, the operating surgeon was blinded to group allocation only until the point of incision; blinding was not maintained during the procedure or for postoperative assessments (operator comfort, complication diagnosis), introducing potential performance and detection bias for these subjective outcomes. Second, randomization lacked allocation concealment and block randomization (see Methods), creating vulnerability to selection bias. Third, the convenience sample of n = 30 was underpowered even for the primary endpoints (post-hoc power approximately 10-16%), and no a priori power calculation was performed. Fourth, all procedures were performed by a single surgeon, which minimized inter-operator variability but limits generalizability across surgeons of differing experience. Fifth, our cohort combined parasymphysis, angle, and body fractures, which may dilute site-specific effects; future trials should consider stratification by fracture site. Sixth, the 10-day follow-up period is insufficient to assess long-term outcomes such as scar quality, cosmetic appearance, and hardware stability; we recommend future trials extend follow-up to at least six to 12 months. Finally, dehiscence and plate exposure were diagnosed by clinical examination alone, without imaging confirmation, and infection was assessed clinically without wound cultures.

Future multi-center randomized controlled trials with n > 100 per arm and stratified permuted-block randomization are needed, following the methodological rigor recently demonstrated by Hawthorne et al. in their single-blind RCT comparing scalpel versus electrocautery for skin incision in thyroidectomy patients [20]. Extended six to 12-month follow-up should incorporate validated patient-reported outcomes [Patient-Reported Outcomes Measurement Information System (PROMIS)], histomorphometric analysis, optical coherence tomography (OCT) thermal profiling, and molecular correlates [interleukin-6 (IL-6), tumor necrosis factor-alpha (TNF-α), vascular endothelial growth factor (VEGF)]. Cost-utility analyses and head-to-head comparisons with ultrasonic devices (Harmonic Scalpel) would forge a robust evidence base for technique selection in maxillofacial surgery.

## Conclusions

In conclusion, this study suggests that the choice between diathermy and a scalpel for mandibular fracture ORIF involves balancing competing priorities, though our findings for incision speed and blood loss should be interpreted cautiously given the limited statistical power of this study (post-hoc power 10-16% for these endpoints). Diathermy showed a non-significant trend toward faster incision and reduced blood loss, but was associated with a longer total operative time, potentially due to challenging tissue handling, and significantly elevated early postoperative pain from thermal injury. By 10 days post-surgery, both techniques achieved equivalent wound healing and comparable safety profiles. Larger, adequately powered trials with stratification by fracture site are needed before firm clinical recommendations can be made regarding the hemostatic and efficiency trade-offs; in the interim, clinicians may weigh the relative importance of minimizing early postoperative pain versus optimizing intraoperative hemostasis based on individual patient factors.
